# The efficacy and safety of ketamine for depression in patients with cancer: A systematic review

**DOI:** 10.1016/j.ijchp.2023.100428

**Published:** 2023-12-15

**Authors:** Leila Azari, Homa Hemati, Ronia Tavasolian, Sareh Shahdab, Stephanie M. Tomlinson, Margarita Bobonis Babilonia, Jeffrey Huang, Danielle B. Tometich, Kea Turner, Heather S.L. Jim, Amir Alishahi Tabriz

**Affiliations:** a University of South Florida Morsani College of Medicine, Tampa, FL, United States; b College of Pharmacy, Tehran University of Medical Sciences, Tehran, Iran; c Department of Clinical Science and Nutrition, University of Chester, England; d University of South Florida Health Libraries Morsani University of South Florida Morsani College of Medicine, Tampa, FL, United States; e Supportive Care Medicine Department, Behavioral Medicine Services, Moffitt Cancer Center, Tampa, FL, United States; f Department of Psychiatry and Behavioral Medicine, University of South Florida Morsani College of Medicine, Tampa, FL, United States; g Department of Oncological Sciences, University of South Florida Morsani College of Medicine, Tampa, FL, United States; h Department of Anesthesiology, Moffitt Cancer Center, Tampa, FL, United States; i Department of Health Outcomes and Behavior, Moffitt Cancer Center, Tampa, FL, United States; j Department of Oncological Sciences, University of South Florida Morsani College of Medicine, Tampa, FL, United States

**Keywords:** Ketamine, Cancer, Depression, Antidepressant, Systematic review

## Abstract

**Background:**

Management of depression in the oncology population includes supportive psychotherapeutic interventions with or without psychotropic medication, which take time to demonstrate effectiveness. Fast-acting interventions, like ketamine, can provide a rapid antidepressant effect; however, there has been limited research on effects of ketamine among cancer patients. The objective of this review is to provide an overview of research on the efficacy and safety of ketamine on depression in patients with cancer.

**Methods:**

We reviewed the published literature in MEDLINE® (via PubMed®), EMBASE, and Scopus from 1 January 1982 to 20 October 2022. We screened the retrieved abstracts against inclusion criteria and conducted a full‐text review of eligible studies. Following extraction of data from included studies, we used a framework analysis approach to summarize the evidence on using ketamine in patients with cancer.

**Results:**

All 5 included studies were randomized clinical trials conducted in inpatient settings in China. In all included studies ketamine was administered intravenously. Three studies used only racemic ketamine, and two studies used both S-ketamine and racemic ketamine. All included studies reported ketamine a tolerable and effective drug to control depression symptoms.

**Conclusion:**

Included studies showed administration of sub-anesthesia ketamine significantly improves postoperative depression among patients with cancer.

## Introduction

Patients with cancer experience an increased risk of depression and suicide compared to the general population. (Amiri & Behnezhad, 2020; Du et al., 2020; Krebber et al., 2014; Lee et al., 2021; Walker et al., 2013) Depression in patients with cancer can negatively impact acceptance and adherence to oncological treatments, extend hospitalization, and increase suicide risk and mortality. (Colleoni et al., 2000; Pitman, Suleman, Hyde & Hodgkiss, 2018; Prieto et al., 2002; Tian, Chen & Hang, 2009; Yousaf, Christensen, Engholm & Storm, 2005) Depression is also associated with greater postoperative pain, higher incidence of postoperative infections, as well as poor health-related quality of life. (Alishahi Tabriz et al., 2023; Wang et al., 2020) Management of depression in the oncology population is challenging because optimal treatment includes a combination of psychotherapeutic interventions with or without psychotropic medication (Mehta & Roth, 2015), which take time to demonstrate effectiveness. For example, supportive psychotherapy interventions can take at least one month to reduce depressive symptoms, and current antidepressants used to manage depression require at least four to six weeks to show their clinical benefit. (Rayner et al., 2010, 2011; Sanacora, Treccani & Popoli, 2012) Providing a fast-acting clinical solution beyond therapeutic intervention for patients with cancer could potentially alleviate added adverse outcomes experienced by this population.

Ketamine, traditionally used as an anesthetic agent, provides a rapid antidepressant effect that can last for one to two weeks after infusion. (Aan Het Rot, Zarate, Charney & Mathew, 2012; Corriger & Pickering, 2019; McGirr et al., 2015) While ketamine has shown significant improvements in clinician-rated measures of depressive severity symptoms among the general population, (McInnes, Qian, Gargeya, DeBattista & Heifets, 2022; Rosenblat et al., 2019; Wilkinson et al., 2018; Xiong et al., 2021) to our knowledge, studies evaluating the efficacy and safety of ketamine in oncology population are limited. This is an important gap to address because of comorbidities, life expectancy, and polypharmacy. Patients with cancer often receive complicated medication regimens to manage their conditions, primarily through oral administration, which often results in significant gastrointestinal symptoms such as nausea, vomiting, diarrhea, constipation, and anorexia. (Andreyev, Davidson, Gillespie, Allum & Swarbrick, 2012; Janelsins et al., 2013) Given the lack of non-enteral formulations for most antidepressant medications, there is a need for alternative formulations that can be administered through routes such as intravenous and intranasal. Ketamine, which is capable of being administered through various routes, including intravenous and intranasal, may potentially decrease side effects and improve symptom burden. The clinical use of ketamine is rapidly evolving, therefore reviewing the evidence available in the literature regarding the efficacy and safety of ketamine for the treatment of depression in the oncology population and identifying potential gaps for future research is needed.

The objective of this systematic literature review is to provide an overview of research on the efficacy and safety of ketamine on depression in patients with cancer. The results of this review can inform future clinical applications of ketamine for depression in patients with cancer.

## Methods

We conducted a systematic literature review according to Preferred Reporting Items for Systematic Reviews and Meta-Analyses guidelines (Appendix A). The study protocol was registered in the International Prospective Register of Systematic Reviews (PROSPERO) (registration number: CRD 42,022,340,316).

### Study inclusion and exclusion criteria

To be included in the review, we required articles to assess the relationship between using ketamine in adult patients with cancer and depression. Additionally, articles were required to be written in the English language, peer-reviewed, and report the results of an empirical study. We excluded articles if the target population was children (younger than 18), and those focused only on molecular aspects of ketamine. A detailed list of inclusion and exclusion criteria can be found in Appendix B.

### Information sources and search strategy

In line with previous research, (Amir Alishahi Tabriz et al., 2022) the literature search strategy was developed by the first author along with a professional medical research librarian. The search was intentionally broad to minimize the risk of overlooking potentially relevant studies. The search strategy was developed for the concepts of cancer and ketamine administration. The search strategies were created using a combination of subject headings and keywords and were used to search MEDLINE® (via PubMed®), EMBASE, and Scopus from 1 January 1982 to 20 October 2022 (40 years of data), when all searches were completed. We also manually scanned the citations of included studies for relevant articles and references from similar systematic reviews in case they were missed during indexing. As we considered only peer-reviewed published studies, gray literature was not included. We applied the Cochrane human studies filter to exclude animal studies and added a systematic review keyword and publication type filter to exclude systematic review articles. The complete strategy for each of the searches can be found in Appendix C.

### Study selection process

Each title and abstract was screened against the eligibility criteria by two researchers. Discrepancies were resolved through discussions between members of each pair and, when necessary, a third team member reviewed the discrepancy until a consensus was reached. To ensure inter-rater reliability of reviews, three iterations of sample reviews were conducted with each person reviewing 30 articles until an average agreement of 83 % was reached. The full-text articles were screened in the same manner.

### Study quality assessment

Two independent researchers assessed the quality of included studies using the NIH Quality Assessment Tool for the controlled intervention studies. ("Study Quality Assessment Tools,") We assigned quality of each study as good, fair, or poor (see Appendix D). Disagreements in the risk of bias scoring were resolved by consensus or by a discussion with a third author.

### Data extraction and analysis

We did not conduct a meta-analysis due to heterogeneity in populations, heterogeneity in how depression was measured, and small sample of the included studies. We used a framework analysis approach to summarize the evidence on using ketamine in patients with cancer. (Ritchie, Lewis, Lewis, Nicholls & Ormston, 2013) The framework analysis approach included five stages (i.e., familiarization, framework selection, indexing, charting, and mapping and interpretation). First, team members read included studies and familiarized themselves with the literature. Second, we identified conceptual frameworks that served as the codes for data abstraction. To describe studies in which researchers have studied administering ketamine in patients with cancer, we used a thematic framework that included publication year, design, outcome(s), type of cancer, objective(s), country, setting, dosage, outcomes, and the relationship between using the ketamine and outcomes. We also collected data on the route of ketamine administration (e.g., infusion, intranasal), and the type of ketamine they used (e.g., S-ketamine (esketamine), R-ketamine (arketamine), R & S Ketamine (racemic ketamine)). Next, pairs of authors completed indexing and charting by placing selected text from included articles into the appropriate cells within our framework. Data from the included studies were extracted into a standardized data extraction form in Microsoft Excel (version 2016). Last, we analyzed extracted data from each cell to describe the studies and findings of using ketamine in patients with cancer.

## Results

### Study selection

**Fig. 1:**
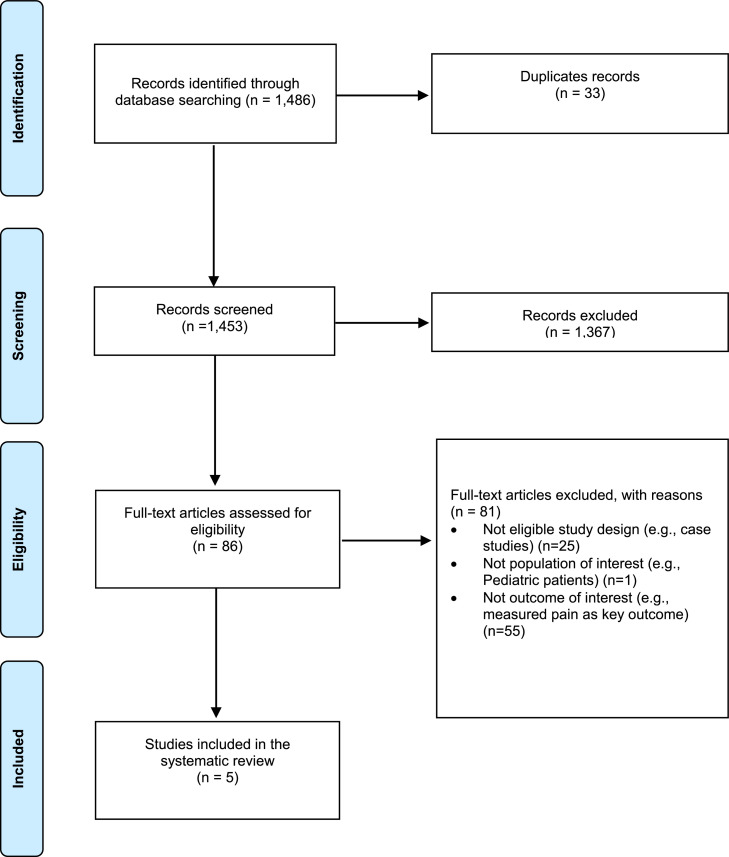
PRISMA Literature Flow Diagram.

The searches in PubMed, Embase, and Scopus yielded 1486 citations. These citations were exported to Endnote (Version 20) and 33 duplicates were removed using the Endnote deduplication feature. This resulted in a total of 1453 unique citations found across all database searches. Titles and abstracts of the 1453 articles were screened; 86 were selected for full-text screening. Of the 86 studies, 81 were excluded at full-text screening or during extraction attempts with the consensus of two coauthors; 5 unique eligible studies were included (Fig. 1).

### Characteristics of included studies

The included studies were conducted between 2014 and 2018. (Fan et al., 2017; Liu et al., 2021; Ren et al., 2022; J. Wang et al., 2020; Xu, Zhan & Chen, 2017) All the included studies were randomized clinical trials and were conducted in inpatient settings (i.e., hospitals) in China. All of the studies targeted surgical patients. The studies covered several cancer types including two focused on breast cancer, (Liu et al., 2021; Xu et al., 2017) one on cervical cancer, (J. Wang et al., 2020) one on colorectal cancer, (Ren et al., 2022) and one study focused on more than one cancer type. (Fan et al., 2017) Characteristics of included studies are shown in Table 1.

**Table 1:** Characteristic of included studies.*.

Citation	Participants (N)	Setting	Recruitment Period	Inclusion/Exclusions criteria
Fan et al. (2017) (1)	Patients with lung (7), gastric (12), bone (7), and pancreas cancer (11)	Huai'an First People's Hospital and Maternal & Child Health Care Hospital of Huai'an City	February 2011 to May 2016	Inclusion criteria: between 18 and 70 years old; first diagnosed as cancer within 3 months; and basic communication capability to complete the interview. Exclusion criteria: diagnosed with cardiorespiratory diseases; drug addiction history or sedative–hypnotic drug(s) use; neuropsychiatric or cognitive diseases or a related treatment history; suicidal attempts or ideation before cancer diagnosis; and family history of psychiatric history
Liu et al. (2021) (2)	Patients with breast cancer (303)	Fengcheng Hospital	June 2017 to June 2018	Inclusion criteria: HAMD-17 8–24 score, and American Society of Anesthesiologists (ASA) score I-II before surgery Exclusion criteria: HAMD score less than or equal to 7 or greater than or equal to 24 before the study, psychiatric disorders such as mania and schizophrenia, and severe liver, renal, cardiovascular, or systematic inflammatory diseases.
Ren et al. (2022) (3)	Patients with colorectal cancer (104)	Gongli hospital	Jan 2015 to October 2017	Inclusion criteria: American Society of Anesthesiologists (ASA) class I–II identification undergoing elective colorectal cancer surgery under general anesthesia for less than 4 h, the incision expected to be more than 10 cm, age between 40 and 70 years with the body mass index (BMI) ranging from 18 to 24 kg m^2^. Exclusion criteria: poor understanding and mental or central nervous system disorders before operation, presence of diabetes and heart disease, hormone therapy during operation, those with ketamine or opioid allergy, presence of severe liver and kidney dysfunction, alcohol addiction or frequent use of sedative and analgesic drugs.
Wang et al. (2020) (4)	Patients with cervical carcinoma (417)	Hospital of Shanghai University	April 2015 to July 2018	Inclusion criteria: Hamilton Rating Scale for Depression scores within 8–24, and American Society of Anesthesiologists score of I-II. Exclusion criteria: Having mental diseases or psychiatric history such as schizophrenia and mania, receiving psychotropic substances, having severe system diseases such as heart, renal and liver diseases.
Xu et al. (2017) (5)	Patients with breast cancer (50)	Hospital of Nanchang University	May 2014 to March 2015	Inclusion criteria: underwent modified radical mastectomy of unilateral breast cancer, age between 30 and 55 years old, have ≥ 5 years of education, American Society of Anesthesiologists I-II grade, HAMD score ≥ 17 points, were married and generational, mainly by the immediate family care after surgery. Exclusion criteria: antidepressant treatment within 2 months, preoperative radiotherapy and chemotherapy treatment, previous personality disorder, mental retardation, brain damage or brain disease, combined with schizophrenia, mania and other mental illness, hyperthyroidism or hypothyroidism, severe cardiovascular disease, diabetes, severe anemia, and heart, lung, liver, kidney function abnormalities, immune system diseases, or the use of drugs affecting the immune system obviously, pregnancy or lactation, a history of illicit drug use (such as marijuana, ecstasy, etc.), participation in other clinical trials, refused to participate.

1. Fan W, Yang H, Sun Y, Zhang J, Li G, Zheng Y, et al. Ketamine rapidly relieves acute suicidal ideation in cancer patients: a randomized controlled clinical trial. Oncotarget. 2017;8(2):2356.

2. Liu P, Li P, Li Q, Yan H, Shi X, Liu C, et al. Effect of Pretreatment of S-Ketamine On Postoperative Depression for Breast Cancer Patients. Journal of Investigative Surgery. 2021;34(8):883–8.

3. Ren Q, Hua L, Zhou X, Cheng Y, Lu M, Zhang C, et al. Effects of a Single Sub-Anesthetic Dose of Ketamine on Postoperative Emotional Responses and Inflammatory Factors in Colorectal Cancer Patients. Frontiers in Pharmacology. 2022;13.

4. Wang J, Wang Y, Xu X, Peng S, Xu F, Liu P**.** Use of various doses of S-ketamine in treatment of depression and pain in cervical carcinoma patients with mild/moderate depression after laparoscopic total hysterectomy. Medical Science Monitor: International Medical Journal of Experimental and Clinical Research. 2020;26:e922028–1.

5. Xu R, Zhan Y, Chen S**.** Effect of intraoperative single administration of sub-anesthesia ketamine on breast cancer patients with depression. BIOMEDICAL RESEARCH-INDIA. 2017;28.

⁎ All the included studies were randomized clinical trials and were conducted in China.

### Quality assessment of studies

The quality of all of the included studies was good (assessed by NIH Quality Assessment Tool for the controlled intervention studies). (Study Quality Assessment Tools) The details of the quality assessment of the included studies are shown in Appendix D.

### Ketamine administration characteristics

In all included studies, ketamine was administered intravenously. Three studies used only racemic ketamine (i.e., racemic ketamine hydrochloride), (Fan et al., 2017; Ren et al., 2022; Xu et al., 2017) and two studies used both S-ketamine and racemic ketamine (both reported that S-ketamine is more effective than racemic ketamine for reducing postoperative depression). (Liu et al., 2021; J. Wang et al., 2020) The dosage of ketamine ranged from 0.1 mg per kg to 0.5 mg per kg. The details of ketamine administration can be found in Table 2.

**Table 2:** Characteristics of Ketamine usage.

Citation	Type of Ketamine	Route of administration	Dosage (including duration)	How long the effects lasted
Fan et al. (2017) (1)	Racemic ketamine hydrochloride	Intravenous	• Ketamine group: 0.5 mg/kg racemic ketamine over 40 min • Control group: 0.05 mg/kg midazolam over 40 min	First three days after surgery
Liu et al. (2021) (2)	Racemic ketamine and S-ketamine	Intravenous	• Control group: 2 ml of normal saline after analgesia induction • Racemic ketamine group: 2 ml of 0.125 mg/kg of racemic ketamine after analgesia induction • S-ketamine group: 2 ml of 0.125 mg/kg of S-ketamine after analgesia induction	One month
Ren et al. (2022) (3)	Ketamine	Intravenous	• Ketamine group 1: 0.1 mg/kg 5 min before operation • Ketamine group 2: 0.2 mg/kg 5 min before operation • Ketamine group 3: 0.3 mg/kg 5 min before operation • Control group: normal saline 5 min before operation	First three days after surgery
Wang et al. (2020) (4)	Racemic ketamine and S-ketamine	Intravenous	• 50 ml 0.5 mg/kg racemic ketamine 1 h after the start of anesthesia • 50 ml 0.5 mg/kg S-ketamine 1 h after the start of anesthesia • 50 ml 0.25 mg/kg S-ketamine 1 h after the start of anesthesia • Control group: 50 ml normal saline 1 h after the start of anesthesia	First three days after surgery
Xu et al. (2017) (5)	Ketamine hydrochloride	Intravenous	• Ketamine group: 0.5 mg/kg ketamine 1 h after the start of anesthesia • Control group: 50 ml of isotonic saline 10 min after the start of anesthesia	First three days after surgery

1. Fan W, Yang H, Sun Y, Zhang J, Li G, Zheng Y, et al. Ketamine rapidly relieves acute suicidal ideation in cancer patients: a randomized controlled clinical trial. Oncotarget. 2017;8(2):2356.

2. Liu P, Li P, Li Q, Yan H, Shi X, Liu C, et al. Effect of Pretreatment of S-Ketamine On Postoperative Depression for Breast Cancer Patients. Journal of Investigative Surgery. 2021;34(8):883–8.

3. Ren Q, Hua L, Zhou X, Cheng Y, Lu M, Zhang C, et al. Effects of a Single Sub-Anesthetic Dose of Ketamine on Postoperative Emotional Responses and Inflammatory Factors in Colorectal Cancer Patients. Frontiers in Pharmacology. 2022;13.

4. Wang J, Wang Y, Xu X, Peng S, Xu F, Liu P**.** Use of various doses of S-ketamine in treatment of depression and pain in cervical carcinoma patients with mild/moderate depression after laparoscopic total hysterectomy. Medical Science Monitor: International Medical Journal of Experimental and Clinical Research. 2020;26:e922028–1.

5. Xu R, Zhan Y, Chen S**.** Effect of intraoperative single administration of sub-anesthesia ketamine on breast cancer patients with depression. BIOMEDICAL RESEARCH-INDIA. 2017;28.

### Outcome measurement

As shown in Table 3, three of included studies used the Hamilton Rating Scale for Depression (HAMD-17), (Liu et al., 2021; J. Wang et al., 2020; Xu et al., 2017) one study measured suicidal ideation using the Beck Scale for Suicidal Ideation (BSI) score and the suicidal section of the Montgomery-Asberg Depression Rating Scale (MADRS-SI), (Fan et al., 2017) and one study measured anxiety and depression using the Hospital Anxiety and Depression Scale (HADS). (Ren et al., 2022) All included studies conducted assessments multiple times, and the assessment time points ranged from one day before the operation to three months after the surgery. As a secondary outcome, four studies measured pain (using Visual Analog Scale (VAS)), (Liu et al., 2021; Ren et al., 2022; J. Wang et al., 2020; Xu et al., 2017) one study measured social support (Xu et al., 2017), one study measured the quality of post-operation recovery (using Quality of Recovery-40 (QoR-40) questionnaire), (Ren et al., 2022) two studies measured serum levels of BDNF and 5-HT, (Liu et al., 2021; J. Wang et al., 2020) and one study measured inflammatory response (e.g., IL-6, IL-8, and TNF-α levels). (Ren et al., 2022) None of the included studies measured comorbid psychotropic medication use (i.e., if ketamine administration changed the medication use among the patients).

**Table 3:** . Included studies outcomes, how and when they measured.

Citation	Main outcome	Secondary outcome(s)	Adverse effects	Assessment Time-points
Fan et al. (2017) (1)	Suicidal ideation measured by the Beck Scale for Suicidal Ideation (BSI) score and suicidal section of the Montgomery-Asberg Depression Rating Scale (MADRS-SI).	• Depression severity measured by MADRS score	Not mentioned	One, three and seven days after operation
Liu et al. (2021) (2)	Depression measured by Hamilton Rating Scale for Depression (HAMD-17).	• Pain status measured by Visual Analog Scale • Serum levels of BDNF and 5-HT was measured by ELISA • Operation time, bleeding volume, and complication rate.	Neither S-ketamine nor racemic ketamine significantly changed the operation time, bleeding volume and complication.	Three days, one week, one month and three months after surgery.
Ren et al. (2022) (3)	Anxiety and depression measured by Hospital Anxiety and Depression Scale.	• The quality of postoperative recovery measured by Quality of Recovery-40 (QoR-40) questionnaire • The levels of IL-6, IL-8, and TNF-α measured by ELISA • Pain measured by Visual Analogue Score (VAS) • Sedation measured by Ramsay Sedation score • Adverse reactions and post-operative complications (cough during extubating, delirium during recovery, sedation within 30 min after extubating, dizziness, nausea, vomiting, diplopia, hallucination, and other adverse reactions)	There were no significant differences in extubation time, postoperative cough, emergence agitation or delirium among the four groups. No dizziness, nausea, vomiting, diplopia, or other adverse reactions were found 30 min after extubation.	One, two and three days after operation
Wang et al. (2020) (4)	Depression measured by Hamilton Rating Scale for Depression (HAMD-17).	• Pain measured by Visual Analogue Score (VAS) • BDNF and 5-HT levels measured by ELISA	No significant difference was observed in operative time, bleeding volume, hospitalization time, or 1-month complication rate	One, two, three, five and seven days after operation
Xu et al. (2017) (5)	Depression measured by Hamilton Rating Scale for Depression (HAMD-17).	• Pain status measured by Visual Analogue Scale/Score (VAS) • The Social Support Scale (SSRS) • Extubating time	There was no significant difference in the incidence of adverse reactions and duration of extubation between the two groups.	One day before operation and one, three and seven days after operation

1. Fan W, Yang H, Sun Y, Zhang J, Li G, Zheng Y, et al. Ketamine rapidly relieves acute suicidal ideation in cancer patients: a randomized controlled clinical trial. Oncotarget. 2017;8(2):2356.

2. Liu P, Li P, Li Q, Yan H, Shi X, Liu C, et al. Effect of Pretreatment of S-Ketamine On Postoperative Depression for Breast Cancer Patients. Journal of Investigative Surgery. 2021;34(8):883–8.

3. Ren Q, Hua L, Zhou X, Cheng Y, Lu M, Zhang C, et al. Effects of a Single Sub-Anesthetic Dose of Ketamine on Postoperative Emotional Responses and Inflammatory Factors in Colorectal Cancer Patients. Frontiers in Pharmacology. 2022;13.

4. Wang J, Wang Y, Xu X, Peng S, Xu F, Liu P**.** Use of various doses of S-ketamine in treatment of depression and pain in cervical carcinoma patients with mild/moderate depression after laparoscopic total hysterectomy. Medical Science Monitor: International Medical Journal of Experimental and Clinical Research. 2020;26:e922028–1.

5. Xu R, Zhan Y, Chen S**.** Effect of intraoperative single administration of sub-anesthesia ketamine on breast cancer patients with depression. BIOMEDICAL RESEARCH-INDIA. 2017;28.

### Efficacy and safety of ketamine

All included studies reported intraoperative single dose ketamine has a rapid antidepressant effect on cancer patients (one day after surgery), but that effect decreases along with time. Four of five included studies reported three days as the duration of ketamine efficacy, (Fan et al., 2017; Ren et al., 2022; J. Wang et al., 2020; Xu et al., 2017) and one study claimed it lasted for a month. (Liu et al., 2021) One study showed the BDNF and 5-HT levels were negatively correlated with the HAMD-17 score, and one study showed a single sub-anesthetic dose of ketamine can reduce the levels of IL-6, IL-8, and TNF-α. (Ren et al., 2022) None of the included studies reported a significant difference in the incidence of adverse events. (Table 4)

**Table 4:** . Objective(s), and conclusion(s) of included studies.   .

Citation	Study objective(s)	Study conclusion
Fan et al. (2017) (1)	To examine the rapid antidepressant effects of single dose ketamine on suicidal ideation and overall depression level in patients with newly diagnosed cancer.	• Ketamine has antidepressant and anti-suicidal effects that were seen as soon as 1 day following administration and typically lasted for at least 3 days • Ketamine is safe and effective for short term use at a sub-anesthetic dose of 0.5 mg/kg over 40 min.
Liu et al. (2021) (2)	To investigate the effect of the pretreatment of S-ketamine on postoperative depression for breast cancer patients with mild/moderate depression.	• S-ketamine is more effective than racemic ketamine for reducing postoperative depression and pain for breast cancer patients. • The BDNF and 5-HT levels were negatively correlated with the HAMD-17 score.
Ren at al. (2020) (3)	To investigate the effect of a single sub-anesthetic dose of ketamine on postoperative anxiety, depression, and inflammatory factors in patients with colorectal cancer.	• A single sub-anesthetic dose (0.3 mg kg-1) of ketamine can significantly improve the postoperative anxiety and depression of colorectal cancer patients and reduce the levels of IL-6, IL-8, and TNF-α.
Wang et al. (2020) (4)	To investigate the effects of various doses of S-ketamine on depression and pain management of cervical carcinoma patients with mild/moderate depression.	• A subanesthetic dose of S-ketamine had better effects on pain and depression than racemic ketamine in cervical carcinoma patients with mild/moderate depression. • High-dose S-ketamine had better efficacy in reducing short-term depression compared with the same dose of racemic ketamine.
Xu et al. (2017) (5)	To observe the effect of single administration of sub - anesthesia ketamine on breast cancer patients with depression.	Intraoperative single administration of sub-anesthesia ketamine has a significant effect on postoperative breast cancer patients with depression, but that effect may decrease along with the time.

1. Fan W, Yang H, Sun Y, Zhang J, Li G, Zheng Y, et al. Ketamine rapidly relieves acute suicidal ideation in cancer patients: a randomized controlled clinical trial. Oncotarget. 2017;8(2):2356.

2. Liu P, Li P, Li Q, Yan H, Shi X, Liu C, et al. Effect of Pretreatment of S-Ketamine On Postoperative Depression for Breast Cancer Patients. Journal of Investigative Surgery. 2021;34(8):883–8.

3. Ren Q, Hua L, Zhou X, Cheng Y, Lu M, Zhang C, et al. Effects of a Single Sub-Anesthetic Dose of Ketamine on Postoperative Emotional Responses and Inflammatory Factors in Colorectal Cancer Patients. Frontiers in Pharmacology. 2022;13.

4. Wang J, Wang Y, Xu X, Peng S, Xu F, Liu P**.** Use of various doses of S-ketamine in treatment of depression and pain in cervical carcinoma patients with mild/moderate depression after laparoscopic total hysterectomy. Medical Science Monitor: International Medical Journal of Experimental and Clinical Research. 2020;26:e922028–1.

5. Xu R, Zhan Y, Chen S**.** Effect of intraoperative single administration of sub-anesthesia ketamine on breast cancer patients with depression. BIOMEDICAL RESEARCH-INDIA. 2017;28.

## Discussion

We conducted this review to summarize the literature about the efficacy and safety of ketamine on depression in patients with cancer. Patients with cancer are at greater risk for depression than the general population and experience negative outcomes as a result of depression (e.g., extended hospitalization, increased postoperative pain and infections, and increased suicide risk and mortality) (Block, 2000; Breitbart et al., 2000; Chachamovich, Fleck, Laidlaw & Power, 2008; Chongpison et al., 2016; Salibasic & Delibegovic, 2018; Y. H. Wang et al., 2020). Despite the severity, our systematic review demonstrates that there have been limited studies evaluating ketamine as a treatment for depression among cancer patients. We only identified five studies (all were conducted in China) that investigate the effects of ketamine on depression among patients with cancer. This finding highlights a need for conducting more robust trials to evaluate safety and efficacy of ketamine on depressive symptoms in patients with cancer.

Our findings showed that ketamine is a safe (no significant adverse events reported) and effective drug to control depression symptoms and correlated symptoms, such as pain. Included studies showed that an intraoperative single administration of sub-anesthesia ketamine significantly improves postoperative depression among patients with cancer. However, included studies showed that the efficacy of ketamine rapidly declined over time. These findings highlight the need for studies that assess the effects of longitudinal usage of ketamine on depression among oncology population. For example, the “S” enantiomer of ketamine (known as esketamine) was approved by the FDA in 2019 as a nasal spray for treatment-resistant depression in adults in conjunction with oral antidepressants. (Bahr, Lopez & Rey, 2019) Future studies may assess continuous esketamine usage in the long-term management of depressive symptoms in patients with cancer.

We found that three of the five included studies compared different types and dosages of ketamine. (Liu et al., 2021; Ren et al., 2022; J. Wang et al., 2020) They showed S-ketamine is more effective than racemic ketamine for reducing postoperative depression, (Liu et al., 2021; J. Wang et al., 2020) and higher doses of ketamine (e.g., 0.5 mg/kg vs 0.25 mg/kg) (J. Wang et al., 2020) had better efficacy in reducing depression. This implies that, instead of ketamine hydrochloride, 0.5 mg/kg (diluted in 0.9 % saline, over 40 min by intravenous pump) of S-ketamine is a more effective choice for management of depression in patients with cancer.

Interestingly, while the participants of four of the five included studies were moderate to severely depressed, (Fan et al., 2017; Liu et al., 2021; J. Wang et al., 2020; Xu et al., 2017) none of the included studies assessed suicidality or comorbid psychotropic medication use (e.g., any changes in the type and/or dose of antidepressants after ketamine administration). Future studies are needed to assess the impact of ketamine administration on the usage of other psychotropic medications in patients with cancer. Additionally, given that cancer patients are at increased risk for suicide, (Fang et al., 2012; Henson et al., 2019; Misono, Weiss, Fann, Redman & Yueh, 2008; Ravaioli et al., 2020; Zaorsky et al., 2019) future studies should assess the effects of ketamine on suicidal ideation among cancer patients.

This review has some limitations. First, our goal was to assess the efficacy and safety of ketamine for treatment of depression among cancer patients; therefore, we excluded studies that only focused on biological aspects of ketamine that may have provided the molecular pathway that explains the relationship between ketamine and depression. Second, we limited our systematic reviews to English-only articles which could result in missing papers published in other languages.

## Conclusion

Despite the ample evidence for treatment of depression in general population, the antidepressant effect of ketamine in the cancer population remains understudied. Current literature shows that administration of intraoperative single-dose of ketamine significantly improves postoperative depression among patients with cancer, however, the efficacy of ketamine declined over time. Future studies are needed to examine ketamine use as a treatment for depression among cancer patients and to assess how longitudinal use of ketamine affects the duration of treatment efficacy.

## Consent for publication

Our manuscript does not contain any identifiable individual-level (patient or clinician) data in any form. We obtained informed consent before each interview.

## Availability of data and material

The data used and/or analyzed during the current study are available from the corresponding author on reasonable request and subject to IRB guidelines.

## Declaration of Competing Interest

The authors declare that they have no competing interests.

## Appendix Supplementary materials

Image, application 1
