# Supplementary material for: The efficacy and safety of ketamine for depression in patients with cancer: A systematic review
Source: Int J Clin Health Psychol. 2023 Dec 15;24(1):100428. doi: 10.1016/j.ijchp.2023.100428 (PMC10764987; doi:10.1016/j.ijchp.2023.100428)
Supplement: Supplementary file 1 [file mmc1.docx]

# **Appendix**

Appendix A. Prisma 2020 Checklist

| **Section and Topic** | **Item #** | **Checklist item** | **Location where item is reported** |
| --- | --- | --- | --- |
| **TITLE** | | |  |
| Title | 1 | Identify the report as a systematic review. | Page 1 |
| **ABSTRACT** | | |  |
| Abstract | 2 | See the PRISMA 2020 for Abstracts checklist. | Page 2 and 3 |
| **INTRODUCTION** | | |  |
| Rationale | 3 | Describe the rationale for the review in the context of existing knowledge. | Page 3 and 4 |
| Objectives | 4 | Provide an explicit statement of the objective(s) or question(s) the review addresses. | Page 5 |
| **METHODS** | | |  |
| Eligibility criteria | 5 | Specify the inclusion and exclusion criteria for the review and how studies were grouped for the syntheses. | Page 5 |
| Information sources | 6 | Specify all databases, registers, websites, organisations, reference lists and other sources searched or consulted to identify studies. Specify the date when each source was last searched or consulted. | Page 5 and 6 |
| Search strategy | 7 | Present the full search strategies for all databases, registers and websites, including any filters and limits used. | Page 5 and 6 |
| Selection process | 8 | Specify the methods used to decide whether a study met the inclusion criteria of the review, including how many reviewers screened each record and each report retrieved, whether they worked independently, and if applicable, details of automation tools used in the process. | Page 6 |
| Data collection process | 9 | Specify the methods used to collect data from reports, including how many reviewers collected data from each report, whether they worked independently, any processes for obtaining or confirming data from study investigators, and if applicable, details of automation tools used in the process. | Page 6 and 7 |
| Data items | 10a | List and define all outcomes for which data were sought. Specify whether all results that were compatible with each outcome domain in each study were sought (e.g. for all measures, time points, analyses), and if not, the methods used to decide which results to collect. | Page 6 and 7 |
|  | 10b | List and define all other variables for which data were sought (e.g. participant and intervention characteristics, funding sources). Describe any assumptions made about any missing or unclear information. | Page 6 and 7 |
| Study risk of bias assessment | 11 | Specify the methods used to assess risk of bias in the included studies, including details of the tool(s) used, how many reviewers assessed each study and whether they worked independently, and if applicable, details of automation tools used in the process. | Page 6 |
| Effect measures | 12 | Specify for each outcome the effect measure(s) (e.g. risk ratio, mean difference) used in the synthesis or presentation of results. | NA |
| Synthesis methods | 13a | Describe the processes used to decide which studies were eligible for each synthesis (e.g. tabulating the study intervention characteristics and comparing against the planned groups for each synthesis (item #5)). | Page 6 and 7 |
|  | 13b | Describe any methods required to prepare the data for presentation or synthesis, such as handling of missing summary statistics, or data conversions. | Page 6 and 7 |
|  | 13c | Describe any methods used to tabulate or visually display results of individual studies and syntheses. | Page 6 and 7 |
|  | 13d | Describe any methods used to synthesize results and provide a rationale for the choice(s). If meta-analysis was performed, describe the model(s), method(s) to identify the presence and extent of statistical heterogeneity, and software package(s) used. | NA |
|  | 13e | Describe any methods used to explore possible causes of heterogeneity among study results (e.g. subgroup analysis, meta-regression). | NA |
|  | 13f | Describe any sensitivity analyses conducted to assess robustness of the synthesized results. | NA |
| Reporting bias assessment | 14 | Describe any methods used to assess risk of bias due to missing results in a synthesis (arising from reporting biases). | NA |
| Certainty assessment | 15 | Describe any methods used to assess certainty (or confidence) in the body of evidence for an outcome. | NA |
| **RESULTS** | | |  |
| Study selection | 16a | Describe the results of the search and selection process, from the number of records identified in the search to the number of studies included in the review, ideally using a flow diagram. | Page 7 and 8 |
|  | 16b | Cite studies that might appear to meet the inclusion criteria, but which were excluded, and explain why they were excluded. | Page 7 and 8 |
| Study characteristics | 17 | Cite each included study and present its characteristics. | Page 8 |
| Risk of bias in studies | 18 | Present assessments of risk of bias for each included study. | Page 8 |
| Results of individual studies | 19 | For all outcomes, present, for each study: (a) summary statistics for each group (where appropriate) and (b) an effect estimate and its precision (e.g. confidence/credible interval), ideally using structured tables or plots. | Page 9-10 |
| Results of syntheses | 20a | For each synthesis, briefly summarise the characteristics and risk of bias among contributing studies. | Page 9 |
|  | 20b | Present results of all statistical syntheses conducted. If meta-analysis was done, present for each the summary estimate and its precision (e.g. confidence/credible interval) and measures of statistical heterogeneity. If comparing groups, describe the direction of the effect. | NA |
|  | 20c | Present results of all investigations of possible causes of heterogeneity among study results. | NA |
|  | 20d | Present results of all sensitivity analyses conducted to assess the robustness of the synthesized results. | NA |
| Reporting biases | 21 | Present assessments of risk of bias due to missing results (arising from reporting biases) for each synthesis assessed. | Page 8 |
| Certainty of evidence | 22 | Present assessments of certainty (or confidence) in the body of evidence for each outcome assessed. | NA |
| **DISCUSSION** | | |  |
| Discussion | 23a | Provide a general interpretation of the results in the context of other evidence. | Page 9-13 |
|  | 23b | Discuss any limitations of the evidence included in the review. | Page 14 |
|  | 23c | Discuss any limitations of the review processes used. | Page 14 |
|  | 23d | Discuss implications of the results for practice, policy, and future research. | Page 9 - 13 |
| **OTHER INFORMATION** | | |  |
| Registration and protocol | 24a | Provide registration information for the review, including register name and registration number, or state that the review was not registered. | Page 5 |
|  | 24b | Indicate where the review protocol can be accessed, or state that a protocol was not prepared. | Page 5 |
|  | 24c | Describe and explain any amendments to information provided at registration or in the protocol. | Page 5 to 9 |
| Support | 25 | Describe sources of financial or non-financial support for the review, and the role of the funders or sponsors in the review. | Page 14 |
| Competing interests | 26 | Declare any competing interests of review authors. | Page 15 |
| Availability of data, code and other materials | 27 | Report which of the following are publicly available and where they can be found: template data collection forms; data extracted from included studies; data used for all analyses; analytic code; any other materials used in the review. | Page 5 |

From: Page MJ, McKenzie JE, Bossuyt PM, Boutron I, Hoffmann TC, Mulrow CD, Shamseer L, Tetzlaff JM, Akl EA, Brennan SE, Chou R. The PRISMA 2020 statement: an updated guideline for reporting systematic reviews. International Journal of Surgery. 2021 Apr 1;88:105906.

For more information, visit: http://www.prisma-statement.org/

Appendix B. PICOTS Table

| **Study Characteristic** | **Inclusion Criteria** | **Exclusion Criteria** |
| --- | --- | --- |
| Population | - Adult patients with cancer | - Children (defined as individuals less than 18 years old) |
| Intervention | - Ketamine administration via oral, IV, IM, subcutaneous, nasal |  |
| Comparison | - Patients with cancer who did not receive Ketamine |  |
| Outcome | - Depression | - Any outcomes not listed |
| Study design | - Randomized trials - Nonrandomized trials - Controlled before-after studies - Cross-sectional - Qualitative studies - Interrupted time-series studies or repeated measures studies - Prospective and retrospective observational studies (i.e., cohort studies, case control studies) | - Descriptive studies with no outcomes data - Case reports and case studies - Modeling studies that used simulated data - Not a clinical study (e.g., editorial, nonsystematic review, letter to the editor) - Measurement or validation studies - Self-described pilot studies without adequate power to assess impact of intervention on outcomes. |
| Time/location | - No limitation |  |
| Publication types | - Full publication in a peer-reviewed journal - Dissertations | - Meeting abstracts, protocols without results, gray literature |

Appendix C. Search Strategy Sample

**The efficacy, and safety of ketamine for depression in patients with cancer**

All studies before duplicate removal: 5,290

All studies after duplicate removal: 2,786

**PubMed Search – Run 10/20/2022.**

Query
(((((Ketamine[Mesh] OR "Receptors, N-Methyl-D- Aspartate"[Mesh])) OR (Ketamin*[Title/Abstract] OR Ketalar[Title/Abstract] OR Ketaject[Title/Abstract] OR Ketanest[Title/Abstract] OR "N Methyl D Aspartate"[Title/Abstract] OR NMDA[Title/Abstract] OR esketamin*[Title/Abstract])) OR (Ketamin*[Other Term] OR Ketalar[Other Term] OR Ketaject[Other Term] OR Ketanest[Other Term] OR "N Methyl D Aspartate"[Other Term] OR NMDA[Other Term] OR esketamin*[Other Term], OR ketofol*[Other Term], arketamine*[Other Term], OR ketalar*[Other Term], OR spravato*[Other Term])) AND ((Neoplasms[mesh] OR neoplasms[tiab] OR neoplasm[tiab] OR neoplasia[tiab] OR neoplasias[tiab] OR neoplastic[tiab] OR dysplastic[tiab] OR dysplasia[tiab] OR dysplasias[tiab] OR "Early Detection of Cancer"[Mesh] OR cancer[tiab] OR cancers[tiab] OR cancerous[tiab] OR malignant[tiab] OR malignancy[tiab] OR malignancies[tiab] OR metastatic[tiab] OR metastasis[tiab] OR metastases[tiab] OR "Biomarkers, Tumor"[Mesh] OR tumor[tiab] OR tumors[tiab] OR tumour[tiab] OR tumours[tiab] OR adenocarcinoma[tiab] OR adenocarcinomas[tiab] OR carcinoma[tiab] OR carcinomas[tiab] OR sarcoma[tiab] OR sarcomas[tiab] OR lymphoma[tiab] OR lymphomas[tiab] OR melanoma[tiab] OR melanomas[tiab] OR leukemia[tiab] OR leukemias[tiab] OR "Cancer Care Facilities"[Mesh] OR "Oncology Service, Hospital"[Mesh] OR oncology[tiab] OR oncologic[tiab] OR chemotherapy[tiab] OR chemotherapies[tiab] OR neoadjuvant therapy[tiab] OR neoadjuvant therapies[tiab] OR chemoradiotherapy[tiab] OR chemoradiotherapies[tiab] OR radioimmunotherapy[tiab] OR radiotherapy[tiab] OR radioimmunotherapies[tiab])) AND (english[Filter])) NOT ((((((Ketamine[Mesh] OR "Receptors, N-Methyl-D- Aspartate"[Mesh])) OR (Ketamin*[Title/Abstract] OR Ketalar[Title/Abstract] OR Ketaject[Title/Abstract] OR Ketanest[Title/Abstract] OR "N Methyl D Aspartate"[Title/Abstract] OR NMDA[Title/Abstract] OR esketamin*[Title/Abstract])) OR (Ketamin*[Other Term] OR Ketalar[Other Term] OR Ketaject[Other Term] OR Ketanest[Other Term] OR "N Methyl D Aspartate"[Other Term] OR NMDA[Other Term] OR esketamin*[Other Term], OR ketofol*[Other Term], arketamine*[Other Term], OR ketalar*[Other Term], OR spravato*[Other Term])) AND ((Neoplasms[mesh] OR neoplasms[tiab] OR neoplasm[tiab] OR neoplasia[tiab] OR neoplasias[tiab] OR neoplastic[tiab] OR dysplastic[tiab] OR dysplasia[tiab] OR dysplasias[tiab] OR "Early Detection of Cancer"[Mesh] OR cancer[tiab] OR cancers[tiab] OR cancerous[tiab] OR malignant[tiab] OR malignancy[tiab] OR malignancies[tiab] OR metastatic[tiab] OR metastasis[tiab] OR metastases[tiab] OR "Biomarkers, Tumor"[Mesh] OR tumor[tiab] OR tumors[tiab] OR tumour[tiab] OR tumours[tiab] OR adenocarcinoma[tiab] OR adenocarcinomas[tiab] OR carcinoma[tiab] OR carcinomas[tiab] OR sarcoma[tiab] OR sarcomas[tiab] OR lymphoma[tiab] OR lymphomas[tiab] OR melanoma[tiab] OR melanomas[tiab] OR leukemia[tiab] OR leukemias[tiab] OR "Cancer Care Facilities"[Mesh] OR "Oncology Service, Hospital"[Mesh] OR oncology[tiab] OR oncologic[tiab] OR chemotherapy[tiab] OR chemotherapies[tiab] OR neoadjuvant therapy[tiab] OR neoadjuvant therapies[tiab] OR chemoradiotherapy[tiab] OR chemoradiotherapies[tiab] OR radioimmunotherapy[tiab] OR radiotherapy[tiab] OR radioimmunotherapies[tiab])) AND (english[Filter])) NOT (("infant"[mesh] OR "child"[mesh] OR "adolescent"[mesh] OR infant[tiab] OR child[tiab] OR adolescent[tiab]) AND (english[Filter])) AND (english[Filter]))

PubMed before duplicate removal: 1,181

PubMed after duplicate removal: 1,179

**Embase Search – Run 10/20/2022.**

('Neoplasm'/exp OR neoplasms:ti,ab OR neoplasm:ti,ab OR neoplasia:ti,ab OR neoplasias:ti,ab OR neoplastic:ti,ab OR dysplastic:ti,ab OR dysplasia:ti,ab OR dysplasias:ti,ab OR 'Early cancer diagnosis'/exp OR cancer:ti,ab OR cancers:ti,ab OR cancerous:ti,ab OR malignant:ti,ab OR malignancy:ti,ab OR malignancies:ti,ab OR metastatic:ti,ab OR metastasis:ti,ab OR metastases:ti,ab OR 'tumor marker'/exp OR tumor:ti,ab OR tumors:ti,ab OR tumour:ti,ab OR tumours:ti,ab OR adenocarcinoma:ti,ab OR adenocarcinomas:ti,ab OR carcinoma:ti,ab OR carcinomas:ti,ab OR sarcoma:ti,ab OR sarcomas:ti,ab OR lymphoma:ti,ab OR lymphomas:ti,ab OR melanoma:ti,ab OR melanomas:ti,ab OR leukemia:ti,ab OR leukemias:ti,ab OR 'Cancer center'/exp OR oncologic:ti,ab OR chemotherapy:ti,ab OR "neoadjuvant therapy":ti,ab OR chemoradiotherapy:ti,ab OR radioimmunotherapy:ti,ab OR radiotherapy:ti,ab OR "neoadjuvant therapies":ti,ab OR chemoradiotherapies:ti,ab OR radioimmunotherapies:ti,ab OR radiotherapies:ti,

Embase after duplicate removal: 672

**CINAHL Search – Run 10/20/2022.**

Query

(MH "Neoplasms+") OR (MH "Oncology") OR (MH "Oncology Care Units") OR (MH "Cancer Patients") OR (MH "Oncologic Care") OR (MH "Chemotherapy, Cancer") OR (neoplasms OR neoplasm OR neoplasia OR neoplasias OR neoplastic OR dysplastic OR dysplasia OR dysplasias OR cancer OR cancers OR cancerous OR malignant OR malignancy OR malignancies OR metastatic OR metastasis OR metastases OR tumor OR tumors OR tumour OR tumours OR adenocarcinoma OR adenocarcinomas OR carcinoma OR carcinomas OR sarcoma OR sarcomas OR lymphoma OR lymphomas OR melanoma OR melanomas OR leukemia OR leukemias OR oncology OR oncologic OR chemotherapy OR chemotherapies OR neoadjuvant therapy OR neoadjuvant therapies OR chemoradiotherapy OR chemoradiotherapies OR radioimmunotherapy OR radiotherapy OR radioimmunotherapies)

CINAHL before duplicate removal: 599

CINAHL after duplicate removal: 265

**Scopus Search – Run 10/20/2022.**

Query

TITLE-ABS(“neoplasms”) OR TITLE-ABS(“neoplasm”) OR TITLE-ABS(“neoplasia”) OR TITLE-ABS(“neoplasias”) OR TITLE-ABS(“neoplastic”) OR TITLE-ABS(“dysplastic”) OR TITLE-ABS(“dysplasia”) OR TITLE-ABS(“dysplasias”) OR TITLE-ABS(“cancer”) OR TITLE-ABS(“cancers”) OR TITLE-ABS(“cancerous”) OR TITLE-ABS(“malignant”) OR TITLE-ABS(“malignancy”) OR TITLE-ABS(“malignancies”) OR TITLE-ABS(“metastatic”) OR TITLE-ABS(“metastasis”) OR TITLE-ABS(“metastases”) OR TITLE-ABS(“tumor”) OR TITLE-ABS(“tumors”) OR TITLE-ABS(“tumour”) OR TITLE-ABS(“tumours”) OR TITLE-ABS(“adenocarcinoma”) OR TITLE-ABS(“adenocarcinomas”) OR TITLE-ABS(“carcinoma”) OR TITLE-ABS(“carcinomas”) OR TITLE-ABS(“sarcoma”) OR TITLE-ABS(“sarcomas”) OR TITLE-ABS(“lymphoma”) OR TITLE-ABS(“lymphomas”) OR TITLE-ABS(“melanoma”) OR TITLE-ABS(“melanomas”) OR TITLE-ABS(“leukemia”) OR TITLE-ABS(“leukemias”) OR TITLE-ABS(“oncology”) OR TITLE-ABS(“oncologic”) OR TITLE-ABS(“chemotherapy”) OR TITLE-ABS(“chemotherapies”) OR TITLE-ABS(“neoadjuvant therapy”) OR TITLE-ABS(“neoadjuvant therapies”) OR TITLE-ABS(“chemoradiotherapy”) OR TITLE-ABS(“chemoradiotherapies”) OR TITLE-ABS(“radioimmunotherapy”) OR TITLE-ABS(“radiotherapy”) OR TITLE-ABS(“radioimmunotherapies”)

Scopus before duplicate removal: 2,079

Scopus after duplicate removal: 670

Appendix D. NIH Quality Assessment Tool for RCTs

**Appendix D-Quality Assessment Tools Used for Assessing the Quality of included Studies**

NIH Quality Assessment Tool for the controlled intervention studies

|  | Fan et al. | Liu et al. | Ren et al. | Wang et al. | Xu et al. |
| --- | --- | --- | --- | --- | --- |
| Was the study described as randomized, a randomized trial, a randomized clinical trial, or an RCT? | Yes | Yes | Yes | Yes | Yes |
| Was the method of randomization adequate (i.e., use of randomly generated assignment)? | Yes | Yes | Yes | Yes | Yes |
| Was the treatment allocation concealed (so that assignments could not be predicted)? | Yes | Yes | Yes | Yes | Yes |
| Were study participants and providers blinded to treatment group assignment? | Yes | Yes | Yes | Yes | Yes |
| Were the people assessing the outcomes blinded to the participants' group assignments? | Yes | Yes | Yes | Yes | Yes |
| Were the groups similar at baseline on important characteristics that could affect outcomes (e.g., demographics, risk factors, co-morbid conditions)? | Yes | Yes | Yes | Yes | Yes |
| Was the overall drop-out rate from the study at endpoint 20% or lower of the number allocated to treatment? | Yes | Yes | Yes | Yes | Yes |
| Was the differential drop-out rate (between treatment groups) at endpoint 15 percentage points or lower? | Yes | Yes | Yes | Yes | Yes |
| Was there high adherence to the intervention protocols for each treatment group? | Yes | Yes | Yes | Yes | Yes |
| Were other interventions avoided or similar in the groups (e.g., similar background treatments)? | Yes | Yes | Yes | Yes | Yes |
| Were outcomes assessed using valid and reliable measures, implemented consistently across all study participants? | Yes | Yes | Yes | Yes | Yes |
| Did the authors report that the sample size was sufficiently large to be able to detect a difference in the main outcome between groups with at least 80% power? | No | No | Yes | No | No |
| Were outcomes reported or subgroups analyzed prespecified (i.e., identified before analyses were conducted)? | Yes | Yes | Yes | Yes | Yes |
| Were all randomized participants analyzed in the group to which they were originally assigned, i.e., did they use an intention-to-treat analysis? | No | Yes | Yes | Yes | Yes |
| **Overall quality rating** | Good | Good | Good | Good | Good |

Overall quality: Good, Fair, Poor

The questions on the form are designed to help assessors focus on the key concepts for evaluating the internal validity of a study. They are not intended to create a list that assessors simply tally up to arrive at a summary judgment of quality.

Internal validity for cohort studies is the extent to which the results reported in the study can truly be attributed to the exposure being evaluated and not to flaws in the design or conduct of the study–in other words, the ability of the study to draw associative conclusions about the effect of the exposures being studied on outcomes. Any such flaws can increase the risk of bias.

Critical appraisal involves considering the risk of potential for selection bias, information bias, measurement bias, or confounding (the mixture of exposures that one cannot tease out from each other). Examples of confounding include co-interventions, differences at baseline in patient characteristics, and other issues throughout the questions above. High risk of bias translates to a rating of poor quality. Low risk of bias translates to a rating of good quality. (Thus, the greater the risk of bias, the lower the quality rating of the study.)

In addition, the more attention in the study design to issues that can help determine whether there is a causal relationship between the exposure and outcome, the higher quality the study. These include exposures occurring prior to outcomes, evaluation of a dose-response gradient, accuracy of measurement of both exposure and outcome, sufficient timeframe to see an effect, and appropriate control for confounding–all concepts reflected in the tool.

Generally, when assessors evaluate a study, assessors will not see a "fatal aw," but assessors will and some risk of bias. By focusing on the concepts underlying the questions in the quality assessment tool, assessors should ask themselves about the potential for bias in the study they are critically appraising. For any box where assessors check "no" they should ask, "What is the potential risk of bias resulting from this aw in study design or execution?" That is, does this factor cause assessors to doubt the results that are reported in the study or doubt the ability of the study to accurately assess an association between exposure and outcome?

The best approach is to think about the questions in the tool and how each one tells assessors something about the potential for bias in a study. The more assessors familiarize themselves with the key concepts, the more comfortable they will be with critical appraisal. Examples of studies rated good, fair, and poor are useful, but each study must be assessed on its own based on the details that are reported and consideration of the concepts for minimizing bias.

# **Tables**

Table 1. Characteristic of included studies.*

| Citation | Participants (N) | Setting | Recruitment Period | Inclusion/Exclusions criteria |
| --- | --- | --- | --- | --- |
| Fan et al. (2017) (1) | Patients with lung (7), gastric (12), bone (7), and pancreas cancer (11) | Huai’an First People’s Hospital and Maternal & Child Health Care Hospital of Huai’an City | February 2011 to May 2016 | Inclusion criteria: between 18 and 70 years old; first diagnosed as cancer within 3 months; and basic communication capability to complete the interview.  Exclusion criteria: diagnosed with cardiorespiratory diseases; drug addiction history or sedative–hypnotic drug(s) use; neuropsychiatric or cognitive diseases or a related treatment history; suicidal attempts or ideation before cancer diagnosis; and family history of psychiatric history |
| Liu et al. (2021) (2) | Patients with breast cancer (303) | Fengcheng Hospital | June 2017 to June 2018 | Inclusion criteria: HAMD-17 8-24 score, and American Society of Anesthesiologists (ASA) score I-II before surgery  Exclusion criteria: HAMD score less than or equal to 7 or greater than or equal to 24 before the study, psychiatric disorders such as mania and schizophrenia, and severe liver, renal, cardiovascular, or systematic inﬂammatory diseases. |
| Ren et al. (2022) (3) | Patients with colorectal cancer (104) | Gongli hospital | Jan 2015 to October 2017 | Inclusion criteria: American Society of Anesthesiologists (ASA) class I–II identification undergoing elective colorectal cancer surgery under general anesthesia for less than 4 h, the incision expected to be more than 10 cm, age between 40 to 70 years with the body mass index (BMI) ranging from 18 to 24 kg m^2^.  Exclusion criteria: poor understanding and mental or central nervous system disorders before operation, presence of diabetes and heart disease, hormone therapy during operation, those with ketamine or opioid allergy, presence of severe liver and kidney dysfunction, alcohol addiction or frequent use of sedative and analgesic drugs. |
| Wang et al. (2020) (4) | Patients with cervical carcinoma (417) | Hospital of Shanghai University | April 2015 to July 2018 | Inclusion criteria: Hamilton Rating Scale for Depression scores within 8-24, and American Society of Anesthesiologists score of I-II.  Exclusion criteria: Having mental diseases or psychiatric history such as schizophrenia and mania, receiving psychotropic substances, having severe system diseases such as heart, renal and liver diseases. |
| Xu et al. (2017) (5) | Patients with breast cancer (50) | Hospital of Nanchang University | May 2014 to March 2015 | Inclusion criteria: underwent modified radical mastectomy of unilateral breast cancer, age between 30-55 years old, have ≥ 5 years of education, American Society of Anesthesiologists I-II grade, HAMD score ≥ 17 points, were married and generational, mainly by the immediate family care after surgery.  Exclusion criteria: antidepressant treatment within 2 months, preoperative radiotherapy and chemotherapy treatment, previous personality disorder, mental retardation, brain damage or brain disease, combined with schizophrenia, mania and other mental illness, hyperthyroidism or hypothyroidism, severe cardiovascular disease, diabetes, severe anemia, and heart, lung, liver, kidney function abnormalities, immune system diseases, or the use of drugs affecting the immune system obviously, pregnancy or lactation, a history of illicit drug use (such as marijuana, ecstasy, etc.), participation in other clinical trials, refused to participate. |

* All the included studies were randomized clinical trials and were conducted in China.

1. Fan W, Yang H, Sun Y, Zhang J, Li G, Zheng Y, et al. Ketamine rapidly relieves acute suicidal ideation in cancer patients: a randomized controlled clinical trial. Oncotarget. 2017;8(2):2356.

2. Liu P, Li P, Li Q, Yan H, Shi X, Liu C, et al. Effect of Pretreatment of S-Ketamine On Postoperative Depression for Breast Cancer Patients. Journal of Investigative Surgery. 2021;34(8):883-8.

3. Ren Q, Hua L, Zhou X, Cheng Y, Lu M, Zhang C, et al. Effects of a Single Sub-Anesthetic Dose of Ketamine on Postoperative Emotional Responses and Inflammatory Factors in Colorectal Cancer Patients. Frontiers in Pharmacology. 2022;13.

4. Wang J, Wang Y, Xu X, Peng S, Xu F, Liu P**.** Use of various doses of S-ketamine in treatment of depression and pain in cervical carcinoma patients with mild/moderate depression after laparoscopic total hysterectomy. Medical Science Monitor: International Medical Journal of Experimental and Clinical Research. 2020;26:e922028-1.

5. Xu R, Zhan Y, Chen S**.** Effect of intraoperative single administration of sub-anesthesia ketamine on breast cancer patients with depression. BIOMEDICAL RESEARCH-INDIA. 2017;28.

Table 2. Characteristics of Ketamine usage.

| Citation | Type of Ketamine | Route of administration | Dosage (including duration) | How long the effects lasted |
| --- | --- | --- | --- | --- |
| Fan et al. (2017) (1) | Racemic ketamine hydrochloride | Intravenous | - Ketamine group: 0.5 mg/kg racemic ketamine over 40 minutes - Control group: 0.05 mg/kg midazolam over 40 minutes | First three days after surgery |
| Liu et al. (2021) (2) | Racemic ketamine and S-ketamine | Intravenous | - Control group: 2 ml of normal saline after analgesia induction - Racemic ketamine group: 2 ml of 0.125 mg/kg of racemic ketamine after analgesia induction - S-ketamine group: 2 ml of 0.125 mg/kg of S-ketamine after analgesia induction | One month |
| Ren et al. (2022) (3) | Ketamine | Intravenous | - Ketamine group 1: 0.1 mg/kg 5 minutes before operation - Ketamine group 2: 0.2 mg/kg 5 minutes before operation - Ketamine group 3: 0.3 mg/kg 5 minutes before operation - Control group: normal saline 5 minutes before operation | First three days after surgery |
| Wang et al. (2020) (4) | Racemic ketamine and S-ketamine | Intravenous | - 50 ml 0.5 mg/kg racemic ketamine 1h after the start of anesthesia - 50 ml 0.5 mg/kg S-ketamine 1h after the start of anesthesia - 50 ml 0.25 mg/kg S-ketamine 1h after the start of anesthesia - Control group: 50 ml normal saline 1h after the start of anesthesia | First three days after surgery |
| Xu et al. (2017) (5) | Ketamine hydrochloride | Intravenous | - Ketamine group: 0.5 mg/kg ketamine 1h after the start of anesthesia - Control group: 50 ml of isotonic saline 10 min after the start of anesthesia | First three days after surgery |

1. Fan W, Yang H, Sun Y, Zhang J, Li G, Zheng Y, et al. Ketamine rapidly relieves acute suicidal ideation in cancer patients: a randomized controlled clinical trial. Oncotarget. 2017;8(2):2356.

2. Liu P, Li P, Li Q, Yan H, Shi X, Liu C, et al. Effect of Pretreatment of S-Ketamine On Postoperative Depression for Breast Cancer Patients. Journal of Investigative Surgery. 2021;34(8):883-8.

3. Ren Q, Hua L, Zhou X, Cheng Y, Lu M, Zhang C, et al. Effects of a Single Sub-Anesthetic Dose of Ketamine on Postoperative Emotional Responses and Inflammatory Factors in Colorectal Cancer Patients. Frontiers in Pharmacology. 2022;13.

4. Wang J, Wang Y, Xu X, Peng S, Xu F, Liu P**.** Use of various doses of S-ketamine in treatment of depression and pain in cervical carcinoma patients with mild/moderate depression after laparoscopic total hysterectomy. Medical Science Monitor: International Medical Journal of Experimental and Clinical Research. 2020;26:e922028-1.

5. Xu R, Zhan Y, Chen S**.** Effect of intraoperative single administration of sub-anesthesia ketamine on breast cancer patients with depression. BIOMEDICAL RESEARCH-INDIA. 2017;28.

Table 3. Included studies outcomes, how and when they measured.

| Citation | Main outcome | Secondary outcome(s) | Adverse effects | Assessment Time-points |
| --- | --- | --- | --- | --- |
| Fan et al. (2017) (1) | Suicidal ideation measured by the Beck Scale for Suicidal Ideation (BSI) score and suicidal section of the Montgomery-Asberg Depression Rating Scale (MADRS-SI). | - Depression severity measured by MADRS score | Not mentioned | One, three and seven days after operation |
| Liu et al. (2021) (2) | Depression measured by Hamilton Rating Scale for Depression (HAMD-17). | - Pain status measured by Visual Analog Scale - Serum levels of BDNF and 5-HT was measured by ELISA - Operation time, bleeding volume, and complication rate. | Neither S-ketamine nor racemic ketamine significantly changed the operation time, bleeding volume and complication. | Three days, one week, one month and three months after surgery. |
| Ren et al. (2022) (3) | Anxiety and depression measured by Hospital Anxiety and Depression Scale. | - The quality of postoperative recovery measured by Quality of Recovery-40 (QoR-40) questionnaire - The levels of IL-6, IL-8, and TNF-α measured by ELISA - Pain measured by Visual Analogue Score (VAS) - Sedation measured by Ramsay Sedation score - Adverse reactions and post-operative complications (cough during extubating, delirium during recovery, sedation within 30 minutes after extubating, dizziness, nausea, vomiting, diplopia, hallucination, and other adverse reactions) | There were no significant differences in extubation time, postoperative cough, emergence agitation or delirium among the four groups. No dizziness, nausea, vomiting, diplopia, or other adverse reactions were found 30 min after extubation. | One, two and three days after operation |
| Wang et al. (2020) (4) | Depression measured by Hamilton Rating Scale for Depression (HAMD-17). | - Pain measured by Visual Analogue Score (VAS) - BDNF and 5-HT levels measured by ELISA | No significant difference was observed in operative time, bleeding volume, hospitalization time, or 1-month complication rate | One, two, three, five and seven days after operation |
| Xu et al. (2017) (5) | Depression measured by Hamilton Rating Scale for Depression (HAMD-17). | - Pain status measured by Visual Analogue Scale/Score (VAS) - The Social Support Scale (SSRS) - Extubating time | There was no significant difference in the incidence of adverse reactions and duration of extubation between the two groups. | One day before operation and one, three and seven days after operation |

1. Fan W, Yang H, Sun Y, Zhang J, Li G, Zheng Y, et al. Ketamine rapidly relieves acute suicidal ideation in cancer patients: a randomized controlled clinical trial. Oncotarget. 2017;8(2):2356.

2. Liu P, Li P, Li Q, Yan H, Shi X, Liu C, et al. Effect of Pretreatment of S-Ketamine On Postoperative Depression for Breast Cancer Patients. Journal of Investigative Surgery. 2021;34(8):883-8.

3. Ren Q, Hua L, Zhou X, Cheng Y, Lu M, Zhang C, et al. Effects of a Single Sub-Anesthetic Dose of Ketamine on Postoperative Emotional Responses and Inflammatory Factors in Colorectal Cancer Patients. Frontiers in Pharmacology. 2022;13.

4. Wang J, Wang Y, Xu X, Peng S, Xu F, Liu P**.** Use of various doses of S-ketamine in treatment of depression and pain in cervical carcinoma patients with mild/moderate depression after laparoscopic total hysterectomy. Medical Science Monitor: International Medical Journal of Experimental and Clinical Research. 2020;26:e922028-1.

5. Xu R, Zhan Y, Chen S**.** Effect of intraoperative single administration of sub-anesthesia ketamine on breast cancer patients with depression. BIOMEDICAL RESEARCH-INDIA. 2017;28.

Table 4. Objective(s), and conclusion(s) of included studies.

| Citation | Study objective(s) | Study conclusion |
| --- | --- | --- |
| Fan et al. (2017) (1) | To examine the rapid antidepressant effects of single dose ketamine on suicidal ideation and overall depression level in patients with newly diagnosed cancer. | - Ketamine has antidepressant and anti-suicidal effects that were seen as soon as 1 day following administration and typically lasted for at least 3 days - Ketamine is safe and effective for short term use at a sub-anesthetic dose of 0.5 mg/kg over 40 minutes. |
| Liu et al. (2021) (2) | To investigate the effect of the pretreatment of S-ketamine on postoperative depression for breast cancer patients with mild/moderate depression. | - S-ketamine is more effective than racemic ketamine for reducing postoperative depression and pain for breast cancer patients. - The BDNF and 5-HT levels were negatively correlated with the HAMD-17 score. |
| Ren at al. (2020) (3) | To investigate the effect of a single sub-anesthetic dose of ketamine on postoperative anxiety, depression, and inflammatory factors in patients with colorectal cancer. | - A single sub-anesthetic dose (0.3 mg kg-1) of ketamine can significantly improve the postoperative anxiety and depression of colorectal cancer patients and reduce the levels of IL-6, IL-8, and TNF-α. |
| Wang et al. (2020) (4) | To investigate the eﬀects of various doses of S-ketamine on depression and pain management of cervical carcinoma patients with mild/moderate depression. | - A subanesthetic dose of S-ketamine had better effects on pain and depression than racemic ketamine in cervical carcinoma patients with mild/moderate depression. - High-dose S-ketamine had better efficacy in reducing short-term depression compared with the same dose of racemic ketamine. |
| Xu et al. (2017) (5) | To observe the effect of single administration of sub - anesthesia ketamine on breast cancer patients with depression. | - Intraoperative single administration of sub-anesthesia ketamine has a significant effect on postoperative breast cancer patients with depression, but that effect may decrease along with the time. |

1. Fan W, Yang H, Sun Y, Zhang J, Li G, Zheng Y, et al. Ketamine rapidly relieves acute suicidal ideation in cancer patients: a randomized controlled clinical trial. Oncotarget. 2017;8(2):2356.

2. Liu P, Li P, Li Q, Yan H, Shi X, Liu C, et al. Effect of Pretreatment of S-Ketamine On Postoperative Depression for Breast Cancer Patients. Journal of Investigative Surgery. 2021;34(8):883-8.

3. Ren Q, Hua L, Zhou X, Cheng Y, Lu M, Zhang C, et al. Effects of a Single Sub-Anesthetic Dose of Ketamine on Postoperative Emotional Responses and Inflammatory Factors in Colorectal Cancer Patients. Frontiers in Pharmacology. 2022;13.

4. Wang J, Wang Y, Xu X, Peng S, Xu F, Liu P**.** Use of various doses of S-ketamine in treatment of depression and pain in cervical carcinoma patients with mild/moderate depression after laparoscopic total hysterectomy. Medical Science Monitor: International Medical Journal of Experimental and Clinical Research. 2020;26:e922028-1.

5. Xu R, Zhan Y, Chen S**.** Effect of intraoperative single administration of sub-anesthesia ketamine on breast cancer patients with depression. BIOMEDICAL RESEARCH-INDIA. 2017;28.
